# Magnetic particle-assisted solid–liquid separation: a rapid and centrifugation-free strategy for the analysis of plant-derived powders and viscous extracts

**DOI:** 10.1038/s41538-026-00980-8

**Published:** 2026-07-10

**Authors:** Dingzhong Wang, Deshou Mao, Yuyang Wang, Bingbiao Cai, Yingjie Fu, Zhiyu Li, Wenjuan Zhang, Weichen Zhang, Hui Xi, Di Chen

**Affiliations:** 1 Key Laboratory of Tobacco Flavor and Fragrance Basic Research of CNTC, Zhengzhou Tobacco Research Institute, Zhengzhou, China; 2 Technology Center, China Tobacco Yunnan Industrial Co., Ltd, Kunming, China; 3 Yunnan Daguan Laboratory, Kunming, China; 4 State Key Laboratory of Cotton Bio-breeding and Integrated Utilization, School of Pharmaceutical Sciences, Zhengzhou University, Zhengzhou, China

**Keywords:** Biochemistry, Biological techniques, Biotechnology, Chemistry, Plant sciences

## Abstract

Plant-derived powders and viscous extracts used in spices, herbal medicines, and foods typically require organic solvent extraction prior to chromatographic analysis, while centrifugation increases operational complexity. Herein, a magnetic particle-assisted solid–liquid separation (MPAS) strategy based on Fe₃O₄ nanoparticles is proposed for rapid, centrifugation-free pretreatment of plant samples. Efficient clarification was achieved using ~50 nm Fe₃O₄ nanoparticles at sample-to-particle mass ratios of 1:15 for powdered samples and 1:5 for viscous extracts, combined with 10 min ultrasonication. The resulting extract compositions were highly consistent with those obtained by conventional centrifugation in both GC–MS and LC–MS analyses, confirming the analytical reliability of MPAS. The method was further applied to monitor drying and heat-treatment processes of herbal medicines and spice powders, enabling dynamic profiling of active constituents and flavor compounds. Overall, MPAS offers a simple and effective centrifugation-free solution for the analysis and quality control of complex plant-derived products.

**Figure:**
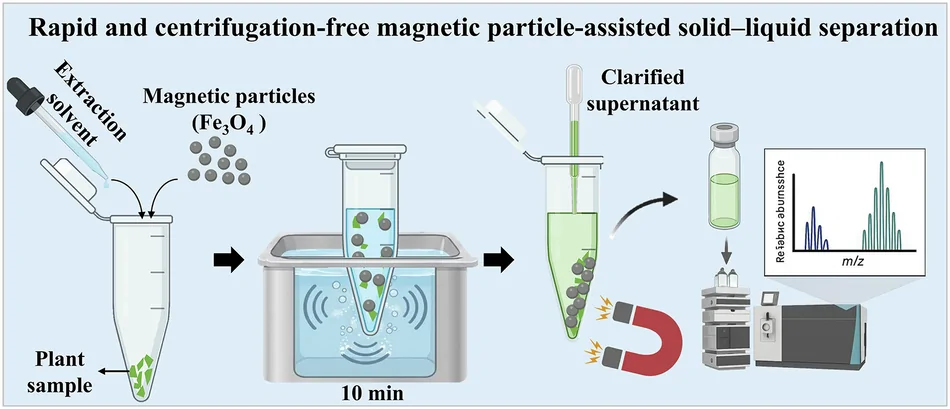

## Introduction

Plant-derived samples, widely used in food, pharmaceutical, and herbal medicine industries, are characterized by their complex chemical composition^1^. These materials often contain a wide variety of bioactive compounds, such as volatile substances, phenolic acids, flavonoids, alkaloids, and high-molecular-weight impurities like polysaccharides^2^. Due to this complexity, effective extraction and pretreatment methods are essential to obtain suitable samples for subsequent analysis^3^.

Techniques such as gas chromatography-mass spectrometry (GC-MS) and liquid chromatography-mass spectrometry (LC-MS) are commonly used for identifying and quantifying these bioactive compounds^4^. Recent studies have demonstrated the broad applicability of GC–MS- and LC–MS-based methods for profiling volatile, semi-volatile, and polar constituents in plant-derived foods, herbal medicines, spices, and natural products^5,6^. In these workflows, solvent extraction combined with ultrasonication, shaking, or maceration is commonly used prior to instrumental analysis^7,8^. However, a subsequent clarification step, typically based on centrifugation, is still required to remove insoluble particles and obtain a supernatant suitable for chromatographic injection^9^. For powdery, fibrous, or viscous plant matrices, this centrifugation-dependent workflow may increase operational complexity, require dedicated equipment, and limit automated or on-site sample processing^10^. Thus, developing a rapid and centrifugation-free clarification strategy remains desirable for complex plant-derived samples.

Recently, numerous studies have focused on the development of self-assembled magnetic adsorbents, which are conveniently synthesized by physically mixing bare magnetic nanoparticles with various adsorbents, without the need for chemical bonding^11,12^. This simple physical mixing approach has proven effective in preparing magnetic adsorbents, such as magnetic graphene and magnetic carbon nanotubes, which have been successfully applied in food, biological, and environmental analysis^13,14^. Notably, Feng’s group has elucidated the principles behind these magnetic adsorbents, highlighting how the physical mixing process and the resulting short-range, non-specific forces significantly enhance separation efficiency and facilitate the formation of magnetic composites^15^. This conceptual advancement reveals that magnetic particles can serve not only as carriers for analyte enrichment but also as functional mediators, enabling the rapid separation of bulk insoluble solids from complex liquid extracts. These insights have inspired the development of novel strategies to address the labor-intensive separation challenges associated with processing plant-derived matrices.

Building on these advancements, this study introduces a magnetic particle-assisted solid–liquid separation (MPAS) strategy utilizing Fe₃O₄ nanoparticles for the rapid, centrifugation-free pretreatment of plant-derived samples. Under ultrasonication, Fe₃O₄ particles associate with insoluble plant solids to form magnetic composites, enabling efficient phase separation via an external magnetic field. The clarification performance was evaluated through visual inspection and turbidity measurements in comparison with conventional centrifugation-based solvent extraction (CSE), followed by a systematic optimization of experimental parameters. To ensure analytical reliability, the chromatographic profiles of representative samples obtained by GC-MS and LC-MS were compared to verify whether the MPAS process would introduce analyte loss. Finally, the method was applied to track dynamic metabolic changes in diverse plant matrices during heat treatment and drying processes, demonstrating its versatility in optimizing the quality and functional properties of plant-derived products.

## Results and discussion

### Initial feasibility assessment

To preliminarily evaluate the feasibility of the proposed MPAS strategy, its separation performance was firstly compared with that of CSE protocol. The primary objective of this assessment was to determine whether MPAS could achieve effective clarification of complex plant-derived suspensions without relying on centrifugation. Representative plant-derived matrices with distinct physical properties were selected for this initial evaluation, including powdered solids (licorice and tobacco powder) and a viscous sample (malt extract). For this assessment, representative plant-derived samples (500 mg) were dispersed in MeOH (25 mL), followed by the addition of Fe₃O₄ magnetic particles (10 g). The suspensions were treated under ultrasonication for 10 min to promote dispersion and interaction between magnetic particles and solid matrices, and subsequently subjected to magnetic separation using an external magnetic field. For comparison, parallel suspensions prepared under identical conditions were processed using a conventional centrifugation procedure at 8000 rpm for 10 min, and the supernatants were carefully collected.

**Fig. 1:**
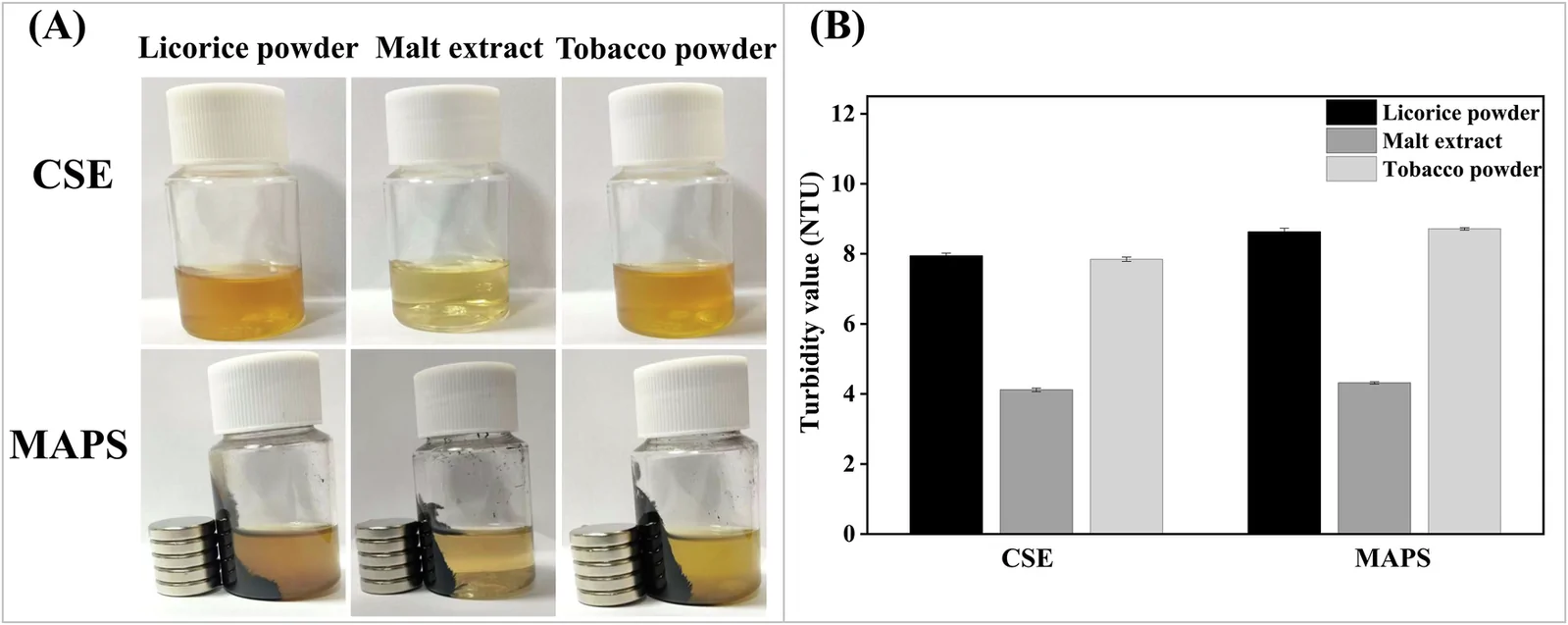
Visual comparison and turbidity assessment of supernatants obtained by conventional CSE and MPAS. **A** photographs of representative supernatants from licorice powder, malt extract, and tobacco powder; **B** turbidity values of the corresponding supernatants.

As schematically illustrated in Fig. 1, suspensions prepared from the different plant matrices were subjected to either centrifugation or MPAS treatment. For the traditional approach, solid–liquid separation was achieved by centrifugation followed by careful collection of the supernatant. In contrast, the MPAS procedure involved the direct addition of Fe₃O₄ particles and magnetic separation using an external magnetic field, without any centrifugation step. Visual inspection of the obtained supernatants (Fig. 1A) showed that MPAS produced clarified solutions comparable to those obtained by centrifugation for all tested sample types. In both approaches, the supernatants appeared transparent and free of visible suspended solids, indicating that MPAS is capable of achieving efficient clarification at a qualitative level.

To further evaluate the clarification performance in a quantitative manner, turbidity measurements were subsequently performed. As shown in Fig. 1B, the results revealed that the turbidity values of supernatants obtained by MPAS were comparable to those obtained using conventional centrifugation, indicating a similar level at removal of fine suspended particulates. This quantitative observation is consistent with the visual comparison and confirms that MPAS can match centrifugation in terms of clarification efficiency.

It should be noted that the ultimate purpose of the clarification step in this study is to ensure analytical compatibility with GC–MS and LC–MS rather than to meet an absolute turbidity threshold. Also, at present, there are no universally accepted regulatory criteria defining turbidity limits for sample pretreatment in analytical workflows. Therefore, method applicability was further evaluated using practical analytical criteria. Specifically, the clarified supernatants obtained by both CSE and MPAS were continuously filtered through 0.22 μm syringe filters with cumulative volumes exceeding 100 mL, and no membrane clogging was observed. In addition, more than 20 consecutive LC–MS injections were performed using MPAS-treated samples without any signs of blockage or abnormal increases in column backpressure; similarly, no issues were encountered during more than 20 consecutive GC–MS injections. These results demonstrate that MPAS is a viable, centrifugation-free alternative for the clarification of complex plant-derived suspensions prior to LC–MS or GC–MS analysis.

From an operational perspective, MPAS and CSE share the same solvent addition and 10 min ultrasonication/extraction steps; therefore, the practical advantage of MPAS is not attributed to a shorter extraction period. Rather, the main difference lies in the clarification step. CSE requires a high-speed centrifuge, tube balancing, and careful aspiration of the supernatant after pelleting, which can disturb the sediment and is not readily compatible with automated workflows. In contrast, MPAS replaces centrifugation with magnetic separation, allowing multiple tubes to be clarified by applying an external magnetic field and making the procedure more compatible with magnetic racks or automated magnetic separation platforms. This feature may be particularly useful when centrifugation is inconvenient or unavailable, such as in on-site pretreatment, parallel or automated sample processing, or processing of viscous and particle-rich plant suspensions.

### Optimization of MPAS parameters

Turbidity value was employed as a quantitative indicator to evaluate the solid-liquid separation efficiency of the MPAS process. All optimization experiments were performed in triplicate, and the turbidity of the supernatant after magnetic separation was measured to reflect the clarity of the solution^16,17^.

For the initial parameter setting, the mass ratio between the sample and Fe₃O₄ particles was defined as 1:20. Fe₃O₄ nanoparticles with an average particle size of 50 nm were employed. Methanol was used as the extraction solvent, and the ultrasonication duration was set to 30 min. These conditions served as the starting point for subsequent optimization experiments.

### Sample-to-Fe₃O₄ mass ratio

**Fig. 2:**
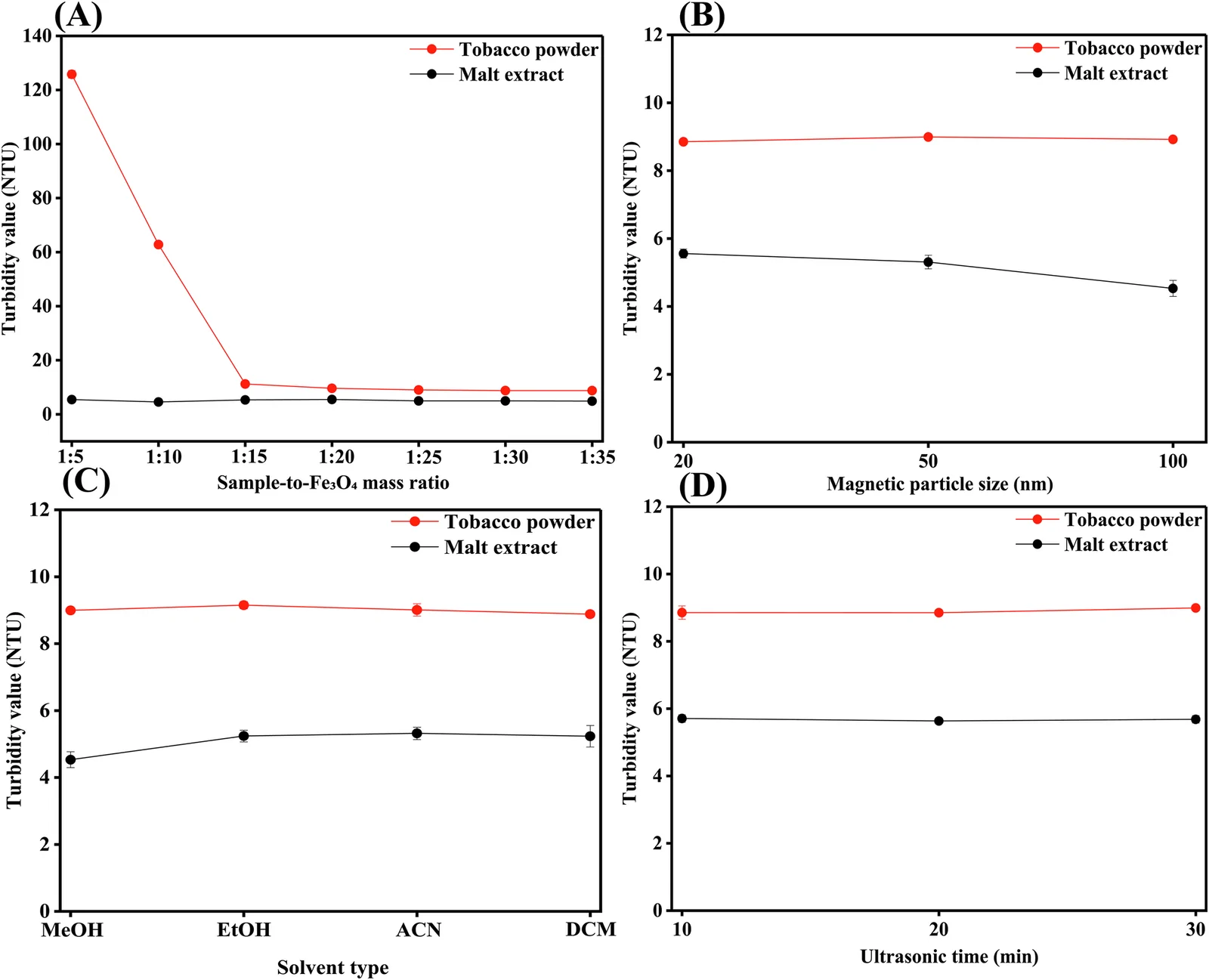
Optimization of key parameters for the MPAS process. **A** sample-to-Fe₃O₄ mass ratio, **B** Fe₃O₄ particle size, **C** solvent type, and **D** ultrasonication time.

The mass ratio between the sample and Fe₃O₄ particles plays a critical role in determining the separation efficiency of the MPAS process. When the sample amount was kept constant, increasing the dosage of Fe₃O₄ particles led to a progressive decrease in the turbidity of the supernatant, indicating enhanced removal of insoluble solids. As shown in Fig. 2A, for powdered samples such as tobacco powder, the turbidity value decreased sharply with increasing Fe₃O₄ dosage and reached a stable plateau at a sample-to-Fe₃O₄ mass ratio of approximately 1:15. This result suggests that, at this ratio, a sufficient number of magnetic particles were available to effectively associate with suspended solid components, thereby achieving efficient magnetic clarification. In contrast, for viscous samples such as malt extract, which contain fewer insoluble particulates, the turbidity was already low at a sample-to-Fe₃O₄ mass ratio of 1:5, and further increases in Fe₃O₄ dosage did not result in a noticeable improvement. Accordingly, mass ratios of 1:15 for powdered samples and 1:5 for viscous samples were selected for subsequent experiments.

### Fe₃O₄ particle size

The influence of Fe₃O₄ particle size on separation performance was further evaluated using magnetic nanoparticles with average diameters of approximately 20, 50, and 100 nm^18^. As shown in Fig. 2B, the turbidity values of the supernatant remained nearly unchanged across the tested particle sizes for both powdered and viscous samples, indicating that Fe₃O₄ particle size has only a limited impact on MPAS efficiency within this size range. This observation demonstrates that MPAS does not require narrowly distributed or highly uniform magnetic nanoparticles, thereby providing considerable flexibility in particle selection and reducing material-related costs. Considering both dispersion stability and magnetic responsiveness, Fe₃O₄ nanoparticles with an average size of approximately 50 nm were employed in subsequent experiments. These results indicate that the MPAS process is not strongly dependent on specially synthesized or highly monodisperse Fe₃O₄ particles. Although in-house synthesis of Fe₃O₄ particles may be further explored in future studies to reduce costs and improve material supply flexibility, the low-cost commercial particles used here were considered practical and suitable for the present MPAS workflow. This also suggests that lower-cost commercial particles with different morphologies or larger particle sizes may be feasible, provided that they offer sufficient magnetic responsiveness and solid-capture ability.

### Solvent type

The selection of the extraction solvent is primarily governed by the physicochemical properties of the target analytes and their compatibility with the analytical platform^19^. In practical applications, solvents commonly used for LC–MS and GC–MS analysis differ in polarity, volatility, and chromatographic compatibility, which may potentially influence the magnetic particle-assisted separation process. As shown in Fig. 2C, the turbidity values of the supernatant obtained using different solvents, including methanol, ethanol, acetonitrile, and dichloromethane, exhibited only minor variations for both powdered (tobacco powder) and viscous (malt extract) samples. These results indicate that solvent type has a negligible effect on the clarification efficiency of MPAS. The consistent turbidity values suggest that the formation of Fe₃O₄–sample composites and the subsequent magnetic separation are largely insensitive to solvent polarity within the tested range.

This solvent tolerance highlights the operational flexibility of MPAS and enables solvent selection to be tailored according to downstream analytical requirements rather than separation efficiency constraints. Based on both solvent compatibility and analytical performance, methanol was selected as the extraction solvent for LC–MS analysis, while ethanol was employed for GC–MS analysis in subsequent experiments.

### Extraction duration

Ultrasonic treatment time is an important parameter influencing particle dispersion and the interaction between Fe₃O₄ particles and suspended solid matrices during the MPAS process. To evaluate its effect on separation efficiency, the ultrasonication duration was systematically varied, and the turbidity of the resulting supernatant was used as the evaluation metric. As shown in Fig. 2D, increasing the ultrasonication time from 10 to 30 min resulted in only minor variations in turbidity for both powdered and viscous samples. The absence of a pronounced decrease in turbidity with prolonged ultrasonication indicates that sufficient dispersion and composite formation are achieved within a relatively short treatment time. Extending the ultrasonication duration beyond this point does not provide additional benefits in terms of clarification efficiency. Considering both operational efficiency and experimental reproducibility, an ultrasonication time of 10 min was selected as the standard extraction condition for the MPAS procedure. Additional short-time trials showed that 3 and 5 min ultrasonication could also achieve effective magnetic phase separation; however, 10 min was retained to ensure sufficient solvent extraction of analytes while maintaining efficient clarification.

### Formation mechanism of Fe₃O₄–plant solid composites

To elucidate the interaction mechanism between magnetic particles and insoluble plant solids during the MPAS process, the morphologies of Fe₃O₄ particles, plant matrices, and their corresponding composites were systematically investigated by scanning electron microscopy (SEM)^20^. Pristine Fe₃O₄ particles and plant matrices were vacuum-dried at 60 °C for 24 h prior to SEM observation. The Fe₃O₄–plant solid composites were obtained following the MPAS procedure and subsequently vacuum-dried under identical conditions to preserve their structural features for microscopic characterization.

**Fig. 3:**
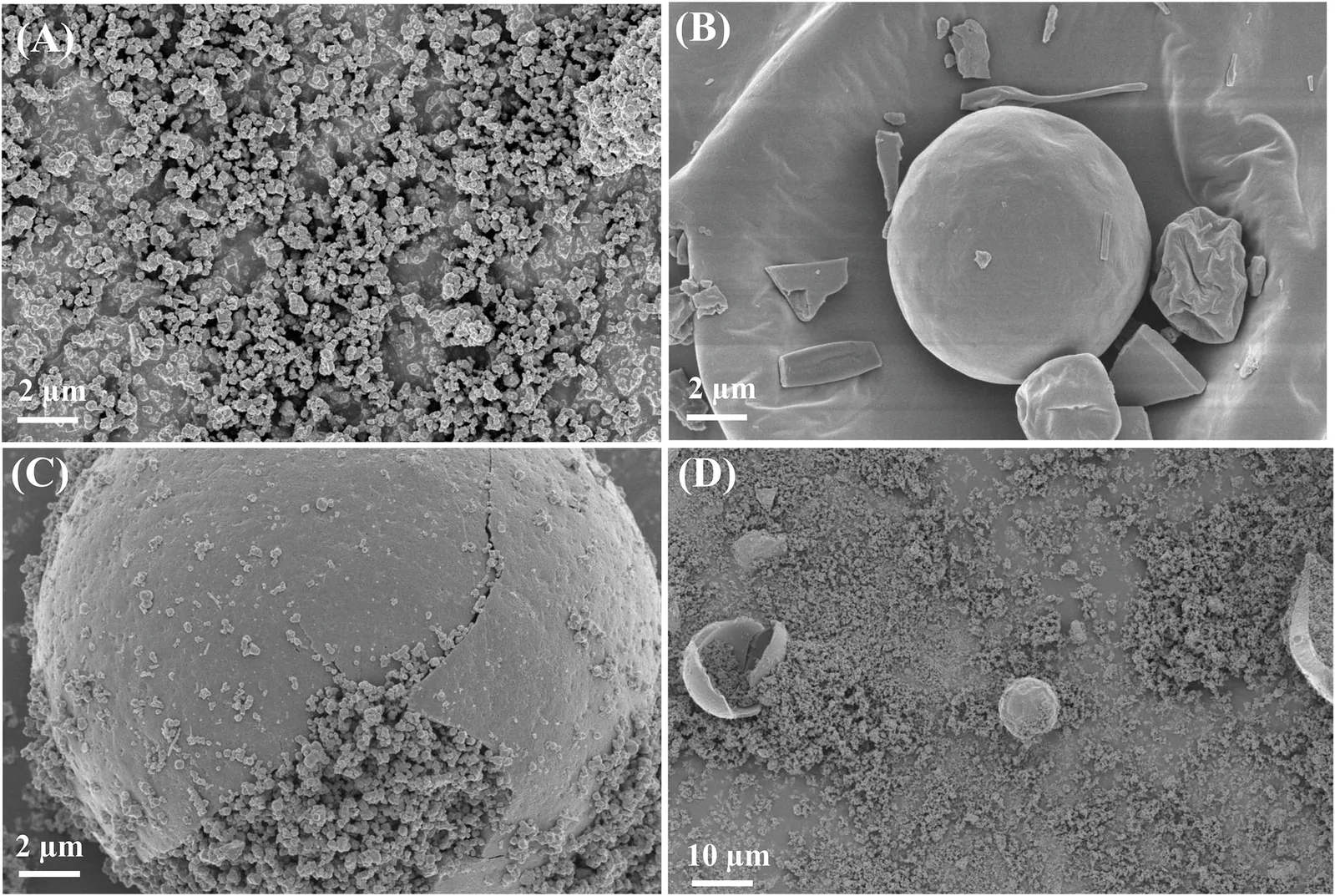
SEM characterization of MPAS-related particles and composites. SEM images of **A** pristine Fe₃O₄ particles, **B** pristine licorice particles, **C** Fe₃O₄/licorice composite at higher magnification, and **D** Fe₃O₄/licorice composite at lower magnification. The composites were obtained using the optimized MPAS procedure and vacuum-dried at 60 °C before SEM observation.

Licorice was first selected as a representative model plant sample for detailed analysis. As shown in Fig. 3A, pristine Fe₃O₄ particles exhibit a granular morphology with relatively uniform particle sizes and rough surfaces. In contrast, licorice particles display compact and comparatively smooth surfaces with limited intrinsic porosity (Fig. 3B). After MPAS, the resulting Fe₃O₄/licorice composites reveal that Fe₃O₄ particles are densely and heterogeneously distributed over the licorice surface, preferentially accumulating at surface irregularities, edges, and micro-wrinkles (Figs. 3C and 3D). Notably, the Fe₃O₄ particles largely retain their original morphology, indicating that the composite formation is dominated by physical association rather than structural deformation or fusion. This observation strongly suggests that the interactions driving composite formation originate from short-range nonspecific forces, such as van der Waals attraction and interfacial contact.

From a mechanistic perspective, these composite structures reflect the dynamic interaction processes occurring in the liquid phase during magnetic particle-assisted separation. Under vortex mixing and ultrasonication, Fe₃O₄ particles are homogeneously dispersed and undergo frequent collisions with suspended plant solids. Once close contact is established, short-range attractive forces enable Fe₃O₄ particles to transiently adhere to the plant surface. Upon application of an external magnetic field, the magnetophoretic motion of Fe₃O₄ particles drives the simultaneous migration of the physically associated plant solids, thereby enabling rapid and efficient solid–liquid separation.

Beyond licorice, the universality of this separation mechanism was further verified using tobacco powder and malt extract as additional plant-derived matrices. As shown in Figs. S1 and S2 (Supporting Information), Fe₃O₄ particles exhibit similar surface coverage and distribution patterns on both tobacco and malt extract solids after MPAS. Despite pronounced differences in morphology and texture among these matrices—ranging from fragmented fibrous structures (tobacco) to relatively compact and viscous solids (malt extract)—the resulting composites consistently display dense and uniform association with Fe₃O₄ particles. These observations collectively demonstrate that the formation of Fe₃O₄–plant solid composites is not sample-specific, but rather governed by general physical interaction principles between magnetic particles and insoluble solid matrices. This universality underpins the robustness of the MPAS strategy and highlights its applicability to diverse plant-derived samples with varying physical properties.

### Evaluation of MPAS for plant-derived sample analysis

The MPAS method was evaluated by comparing its performance with conventional CSE for the analysis of complex plant-derived samples, including both powder-based and viscous extracts commonly encountered in food, herbal medicine, and natural product analysis. The samples included grape extract, malt extract, licorice powder, jujube extract, and tobacco powder, among others. The evaluation was performed using both GC-MS and LC-MS platforms to assess overall chromatographic consistency and relative signal responses between the two pretreatment methods.

**Fig. 4:**
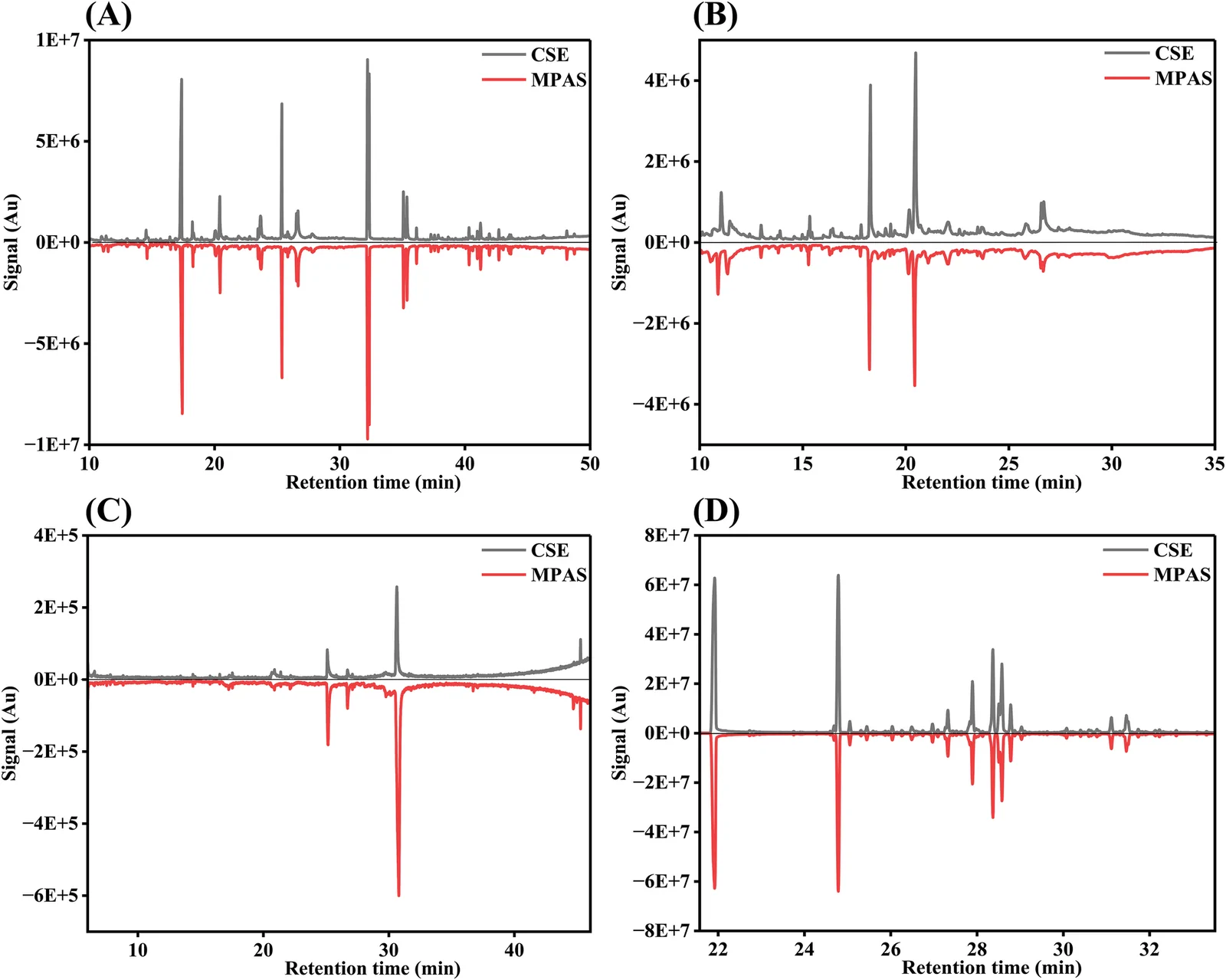
TICs of plant-derived sample extracts analyzed by GC–MS. **A** malt extract, **B** jujube extract, **C** licorice powder, and **D** cinnamon powder. Ethanol was used as the extraction solvent, and MPAS was performed with 10 min ultrasonication followed by magnetic separation.

For GC-MS analysis, Fig. 4 presents the chromatograms of the MPAS-treated samples, compared with those treated using CSE. The total ion chromatography (TIC) showed that the peak distributions and overall signal characteristics were highly consistent between both methods. For example, in the analysis of malt extract (Fig. 4A), jujube extract (Fig. 4B), licorice powder (Fig. 4C), and cinnamon powder (Fig. 4D), the MPAS-treated samples exhibited similar peak positions and intensities as those obtained by CSE, demonstrating that MPAS provides comparable extraction efficiency. The results indicated that both methods yield similar chromatographic profiles, further validating MPAS as an effective alternative to CSE. Further validation of the GC-MS results using Fig. S3 confirmed the reproducibility and reliability of MPAS across various plant-derived matrices, including dandelion extract, grape extract, danshen powder, nutgrass powder, scutellaria baicalensis powder, and tobacco powder. This further reinforces the method’s efficacy as a viable alternative to CSE.

**Fig. 5:**
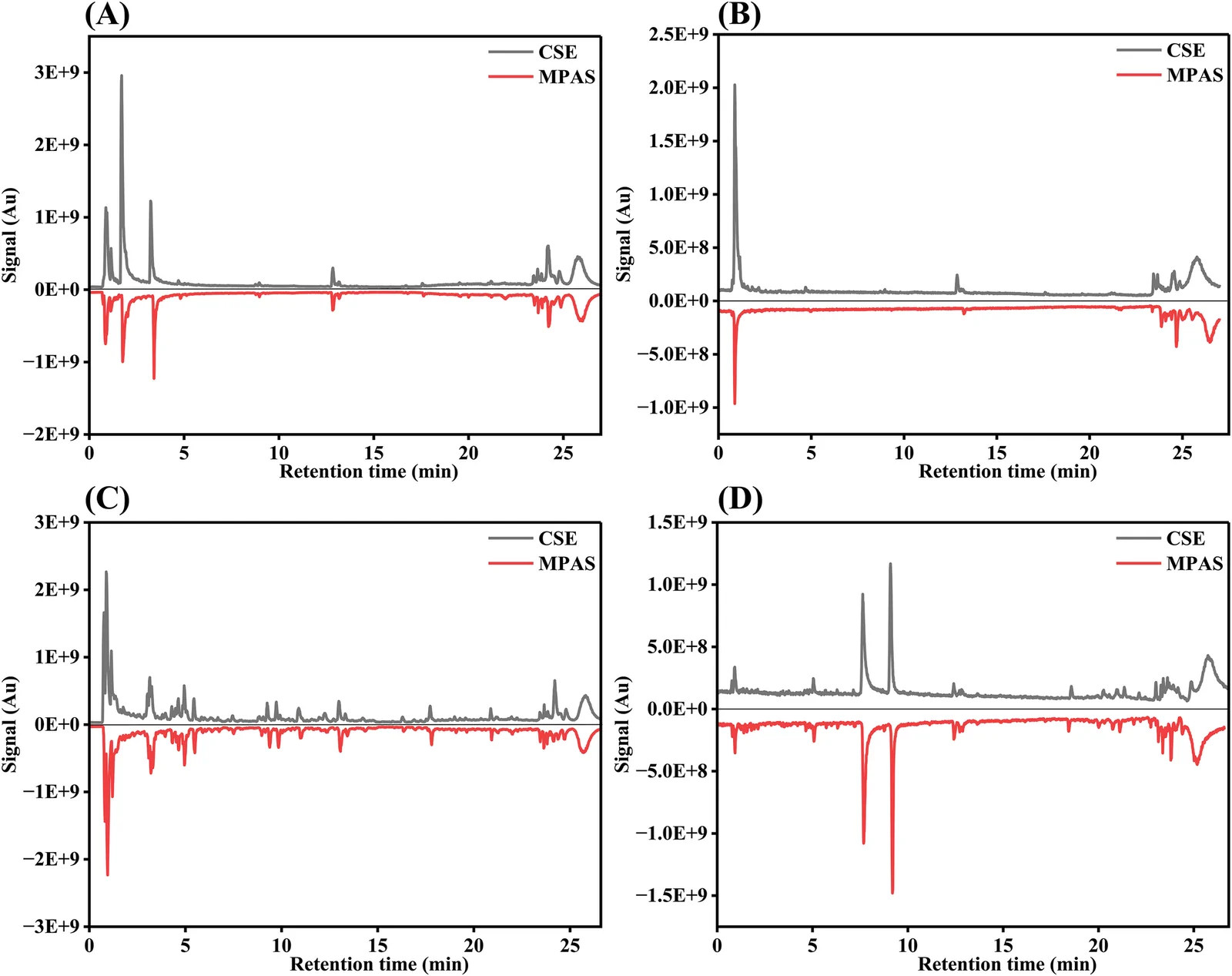
TICs of plant-derived sample extracts analyzed by high-resolution LC–MS in positive ion mode. **A** malt extract, **B** jujube extract, **C** licorice powder, and **D** cinnamon powder. Methanol was used as the extraction solvent, and MPAS was performed with 10 min ultrasonication followed by magnetic separation.

In LC-MS analysis, the chromatograms from MPAS-treated samples were also compared with those from CSE-treated samples, as shown in Fig. 5. The TICs revealed that MPAS produced chromatographic profiles nearly identical to those obtained from CSE, with consistent peak positions and intensities. This demonstrates that MPAS provides similar extraction efficiency to CSE across different plant extracts and powders. To further verify the robustness of MPAS, additional plant-derived samples, including licorice powder, tobacco powder, and cinnamon powder, were analyzed. The results presented in Fig. S4 showed consistent chromatographic profiles across these samples, further confirming the method’s reliability in various sample types.

Overall, the MPAS method was found to be a reliable and efficient alternative to CSE for the solid-liquid separation of plant-derived samples. Both GC-MS and LC-MS analyses demonstrated that MPAS provided chromatographic profiles comparable to those obtained by CSE, offering similar extraction efficiencies. The simplicity of the MPAS method, reduced dependence on centrifugation, and consistent performance across a wide range of complex plant matrices make it a useful tool for parallel or automated sample pretreatment and quality control in natural product research.

### Monitoring metabolite changes during plant sample heat processing

Plant-derived raw materials, such as nutgrass, cinnamon, and malt extract, are frequently subjected to drying processes during the preparation of granules, instant powders, or capsule formulations. These drying processes are often accompanied by heat treatment, which may lead to the degradation or transformation of certain active compounds. Such changes can have significant effects on the quality and stability of the final product. Additionally, in food processing, these plant materials are commonly used in baked products, where high-temperature processing can notably impact the stability of bioactive compounds and the retention of flavor components. Therefore, it is essential to monitor and understand these metabolic changes during processing to optimize conditions that preserve both functional and sensory properties^21,22^.

Herein, MPAS was applied to track the dynamic changes of metabolites in plant samples during heat treatment. The experiment involved exposing plant materials, including cinnamon powder, malt extract, nutgrass powder, dandelion extract, and tobacco powder, to three different temperatures (40, 60, and 80 °C) and sampling at various time intervals (0, 2, 4, 6, and 8 h). Subsequently, the samples were processed using MPAS, and the clarified supernatants were analyzed by GC-MS and LC-MS to evaluate the changes in metabolite abundance. The major chemical constituents identified by GC-MS and LC-MS are summarized in Tables S1 and S2, respectively, providing a comprehensive overview of the detected compounds and their corresponding properties.

**Fig. 6:**
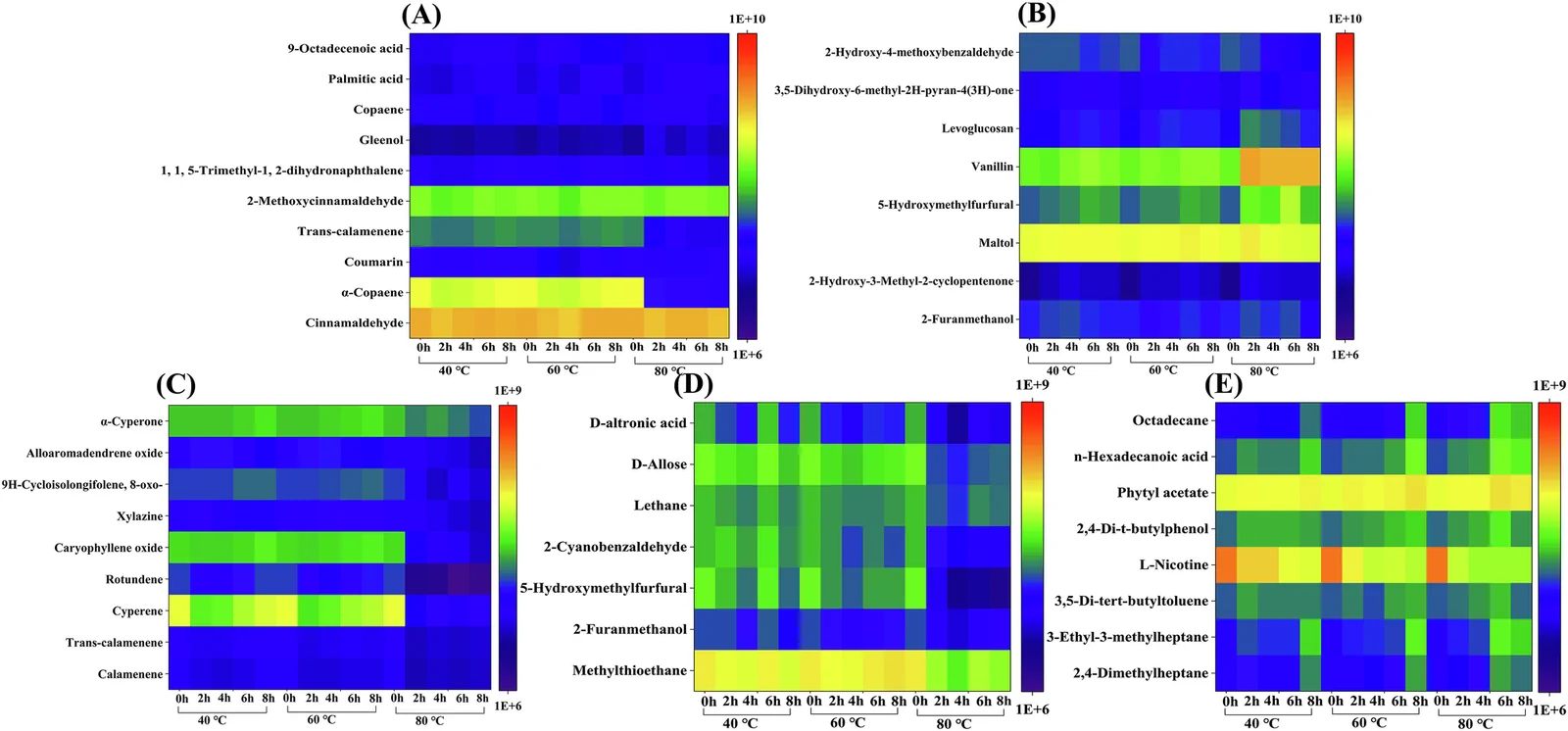
Heatmap of metabolite variation in different plant samples at different temperatures and time points using GC-MS analysis. This figure shows the relative abundance changes of metabolites in different plant samples: **A** cinnamon powder, **B** malt extract, **C** nutgrass powder, **D** dandelion extract, and **E** tobacco powder, at various temperatures (40, 60, 80 °C) and time points (0, 2, 4, 6, 8 h).

**Fig. 7:**
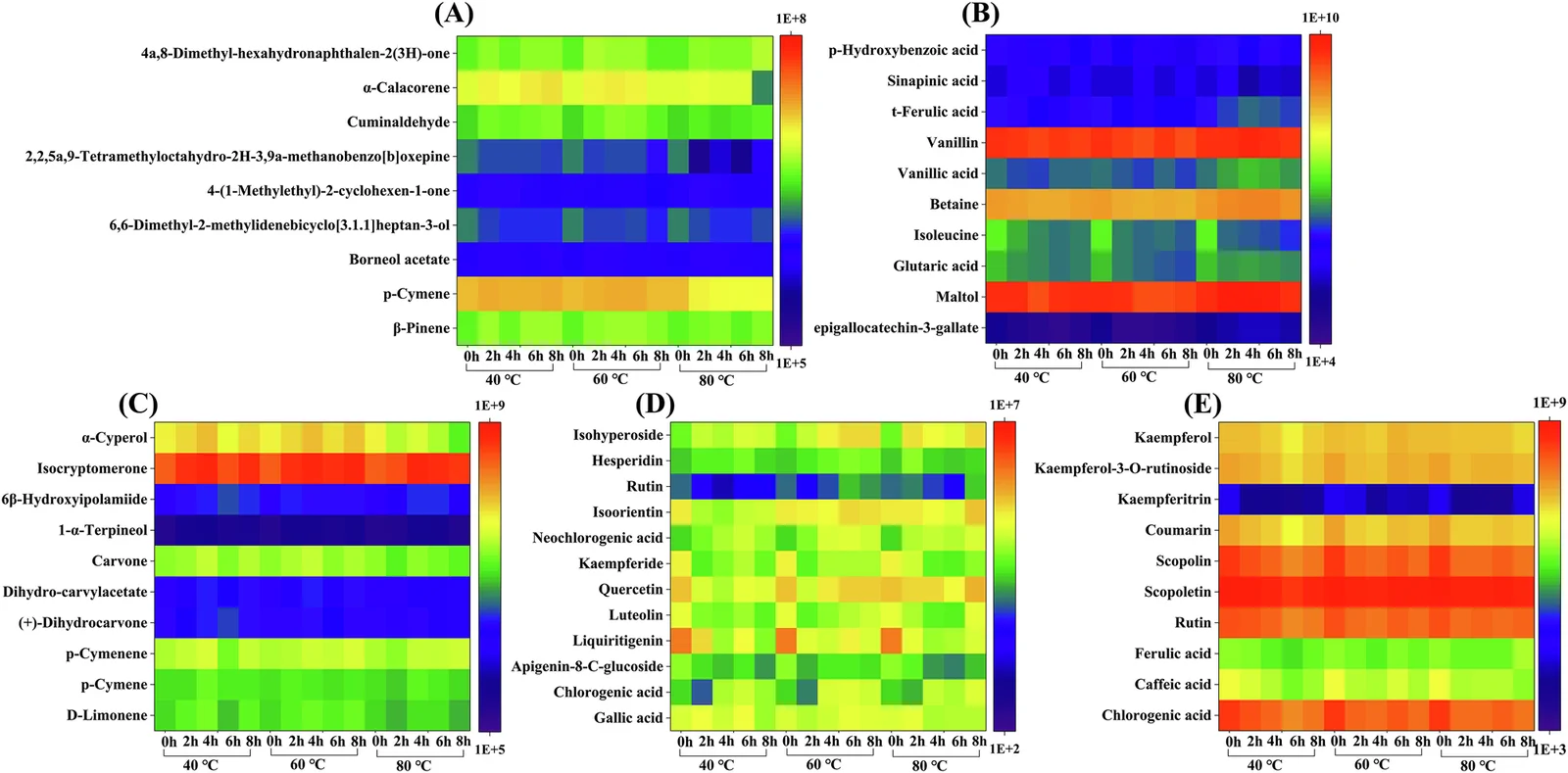
Heatmap of metabolite variation in different plant samples at different temperatures and time points using LC-MS analysis. This figure shows the relative abundance changes of metabolites in different plant samples: **A** cinnamon powder, **B** malt extract, **C** nutgrass powder, **D** dandelion extract, and **E** tobacco powder, at various temperatures (40, 60, 80 °C) and time points (0, 2, 4, 6, 8 h).

In cinnamon powder (Figs. 6A and Fig. 7A), both GC-MS and LC-MS results revealed significant decreases in the abundance of several volatile aromatic compounds, such as trans-calamenene, α-copnene, α-calacorene, and p-cymene, at 80 °C^23^. These compounds are highly volatile, and high temperatures likely accelerated their vaporization or degradation, leading to a marked reduction in their residual amounts in the samples. This decrease aligns with the findings in both chromatographic techniques, suggesting that heat treatment significantly impacts the retention of these compounds. The reduction of volatile components could alter the aroma and flavor profile of cinnamon powder, which is crucial for both food and medicinal applications.

In contrast, the analysis of malt extract (Figs. 6B and 7B) by both GC-MS and LC-MS showed a marked increase in the abundance of vanillin at 80 °C. This phenomenon is likely due to the reduction of moisture content during heating, leading to a relative concentration effect. The increase in vanillin abundance under high-temperature conditions suggests that high-temperature processing can enhance the concentration of certain flavor compounds rather than simply causing their degradation. This highlights the dual effects of high-temperature processing, which can both degrade and concentrate specific compounds, ultimately influencing the final flavor and functional properties^24,25^.

For nutgrass powder (Figs. 6C and 7C), both GC-MS and LC-MS showed a significant reduction in the abundance of multiple sesquiterpenes, such as α-cyperone, caryophyllene oxide, and cyperene, at 80 °C. These compounds are known to be highly volatile, and the elevated temperature likely accelerated their evaporation. The decrease in sesquiterpenes at high temperatures is consistent with the general volatility of these constituents, which are often used for their aromatic properties in essential oils and herbal medicines. This reduction could influence the therapeutic or sensory qualities of nutgrass powder, particularly in medicinal and food applications where these volatile compounds are key components^26^.

In dandelion extract (Figs. 6D and 7D), both GC-MS and LC-MS revealed a decrease in the abundance of D-allose and methylthioethane at 80 °C. Methylthioethane, a sulfur-containing compound with high vapor pressure, is highly volatile and readily evaporates at elevated temperatures. D-allose, a sugar, is less volatile but may undergo thermal degradation or structural transformation, leading to its reduced presence in the sample. Additionally, LC-MS analysis showed an increase in polyphenolic compounds, such as gallic acid and neochlorogenic acid, at 80 °C. This increase could be due to reduced water content during heating, leading to a relative concentration effect, or due to the release of bound polyphenols during thermal treatment. These findings suggest that high-temperature treatment may influence both the volatility and stability of various chemical constituents in dandelion extract.

In tobacco powder (Figs. 6E and 7E), GC-MS and LC-MS analyses indicated a significant decrease in nicotine content as the temperature increased. Nicotine, a volatile alkaloid, is more easily released from the solid matrix into the gas phase at higher temperatures, leading to its reduced content in the sample. This observation is consistent with known processes in tobacco baking or combustion, where nicotine evaporates at high temperatures. Additionally, in LC-MS, caffeic acid abundance gradually decreased with rising temperature and extended heating duration. This suggests that caffeic acid may undergo degradation or transformation at high temperatures, forming other compounds and leading to reduced detectable levels. These changes in nicotine and caffeic acid suggest that high-temperature processing has a significant impact on alkaloid stability in tobacco powder.

Taken together, the GC-MS and LC-MS results indicate that high-temperature processing has profound effects on both volatile and polar metabolites in plant samples. Volatile compounds such as terpenes and aromatic compounds are more prone to evaporation or degradation under high-temperature conditions, while some polar compounds may undergo concentration effects or structural transformations, leading to changes in their apparent abundance. The observed variations in metabolite abundances across different plant samples highlight the complexity of heat treatment effects, which can both degrade and enhance certain compounds, thereby influencing the flavor, aroma, and functional properties of the processed plant materials. The newly developed centrifugation-free pretreatment method demonstrated stable and sensitive detection of dynamic metabolite changes under different processing conditions. This validates its applicability and reliability in the analysis of complex matrices^27^.

Despite these advantages, the reusability of Fe₃O₄ particles should also be considered. In the current MPAS workflow, Fe₃O₄ particles were used as single-use magnetic separation aids. After separation, they became physically associated with plant-derived powders, fibers, colloids, and other insoluble residues, making complete recovery and direct reuse impractical. However, this does not substantially compromise the practical applicability of MPAS because low-cost commercial Fe₃O₄ particles can be used. Possible analyte adsorption should also be considered when applying MPAS. The surface of Fe₃O₄ particles may interact with some metabolites through weak interactions, such as electrostatic attraction, hydrogen bonding, coordination, or other nonspecific interactions, which could lead to partial retention of certain analytes on the magnetic particle-solid composites. In this study, the highly similar GC-MS and LC-MS chromatographic profiles obtained by MPAS and conventional CSE suggest that no obvious broad or selective loss occurred for the representative plant matrices evaluated. However, chromatographic profile comparison alone cannot completely exclude adsorption of individual compounds. Therefore, for targeted applications involving specific marker compounds, analyte recovery and possible adsorption loss should be further evaluated using appropriate standards or spiked samples.

Collectively, the proposed MPAS method presents a promising, efficient, and user-friendly alternative to conventional CSE for plant-derived sample analysis. By leveraging Fe₃O₄ nanoparticles, MPAS eliminates the need for centrifugation, significantly simplifying the sample pretreatment process, while maintaining analytical reliability. Both GC-MS and LC-MS analyses revealed that MPAS provided chromatographic profiles consistent with those obtained by traditional methods, demonstrating its comparable extraction performance across a wide range of plant matrices, including powdered and viscous samples. Furthermore, MPAS was successfully applied to monitor dynamic metabolite changes during heat treatment and drying processes, offering valuable insights into the thermal degradation and concentration effects of bioactive compounds. At the same time, several practical limitations should be considered during application, including single-use consumption and handling of magnetic particles, as well as the possible adsorption of individual analytes in targeted applications. Overall, the method’s simplicity, low cost, reduced dependence on centrifugation, and broad compatibility with plant-derived matrices underscore its potential for parallel or automated sample pretreatment, quality control, and process monitoring in food and medicinal plant industries. Future studies will focus on expanding its application to additional sample types, improving particle handling, and integrating MPAS with validated quantitative workflows for selected marker compounds.

## Methods

### Chemicals and reagents

Three size variants of Fe₃O₄ nanoparticles with average diameters of approximately 20, 50, and 100 nm were purchased from Shanghai MCC New Materials Co., Ltd. (Shanghai, China) and used as received without further purification. At the time of purchase, the approximate prices of the 20, 50, and 100 nm commercial Fe₃O₄ particles were 75, 52, and 47 RMB per 100 g, respectively, corresponding to approximately 10.4, 7.2, and 6.5 USD per 100 g. LC–MS-grade methanol (MeOH), ethanol (EtOH), acetonitrile (ACN), dichloromethane (DCM), and formic acid (FA) were obtained from Thermo Fisher Scientific (Waltham, MA, USA) and were of analytical quality suitable for chromatographic and mass spectrometric analysis. Ultrapure water (H₂O) was produced using a Milli-Q water purification system (18.2 MΩ·cm, Millipore, Billerica, MA, USA) and was used for the preparation of mobile phases of LC-MS.

### Sample sources

Malt extract (*Hordeum vulgare* L.), jujube extract (*Ziziphus jujuba* Mill.), dandelion extract (*Taraxacum mongolicum* Hand.-Mazz.), grape extract (*Vitis vinifera* L.), licorice powder (*Glycyrrhiza uralensis* Fisch.), and tobacco powder (*Nicotiana tabacum* L.) were provided by the Zhengzhou Tobacco Research Institute (Zhengzhou, China). Scutellaria baicalensis powder (*Scutellaria baicalensis* Georgi), cinnamon powder (*Cinnamomum cassia* L. J.Presl), nutgrass powder (*Cyperus rotundus* L.), and danshen powder (*Salvia miltiorrhiza* Bunge) were purchased from a local market in Zhengzhou, China. These samples were selected to represent both powdered and viscous plant-derived matrices commonly encountered in food, herbal medicine, and natural product analysis. All samples were stored in a cool, well-ventilated environment at approximately 15 °C prior to analysis.

### Conventional centrifugation-based solvent extraction

**Fig. 8:**
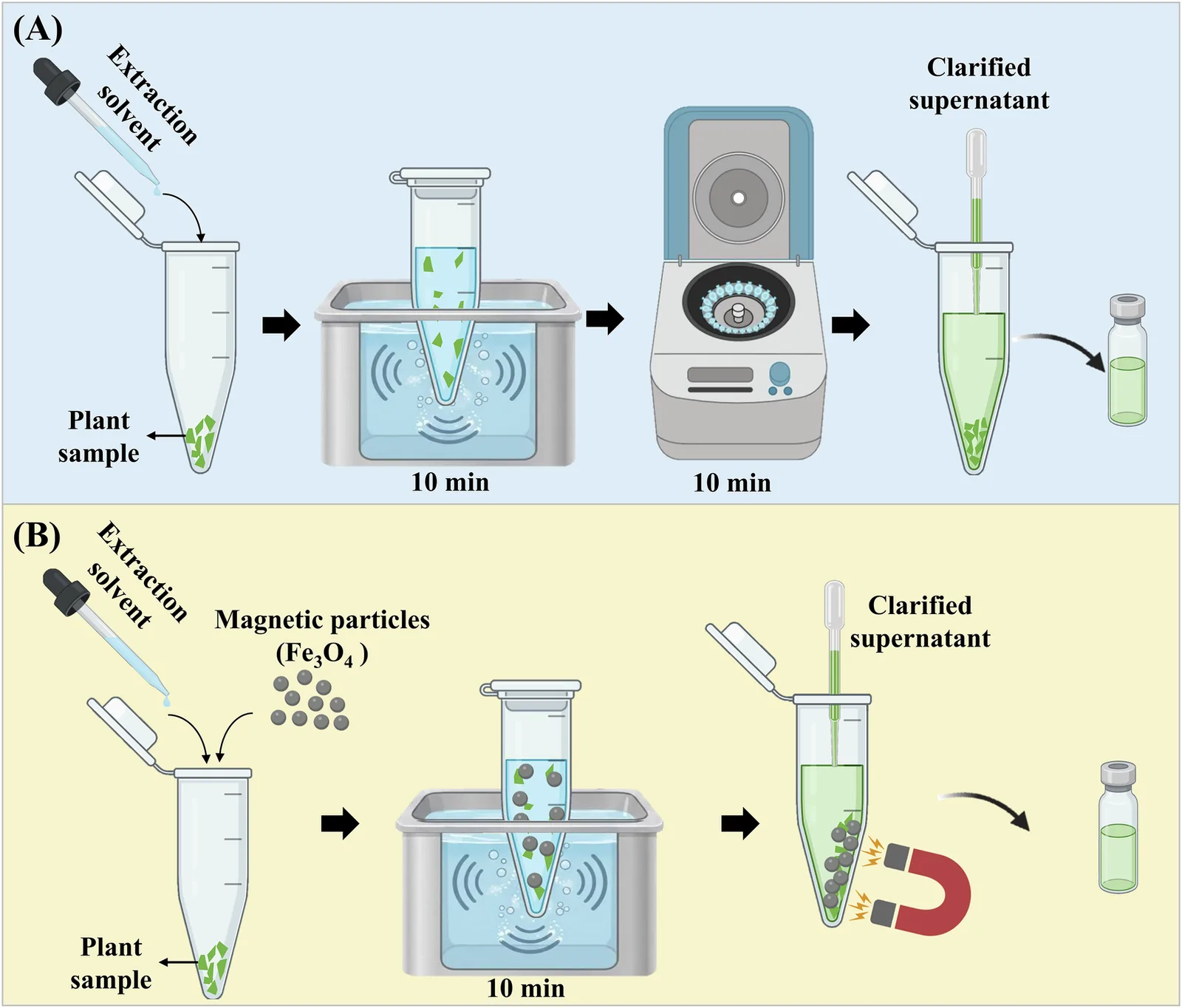
Comparison of conventional and magnetic particle-assisted sample separation workflows. **A** Conventional centrifugation-based solvent extraction and **B** magnetic particle-assisted solid–liquid separation (MPAS) procedures for plant-derived samples prior to GC–MS or LC–MS analysis.

Conventional CSE was employed as a reference method for sample preparation (Fig. 8A). For GC–MS analysis, EtOH was used as the extraction solvent for all samples except tobacco powder, which was extracted using DCM at a concentration of 50 mg/mL. For LC–MS analysis, MeOH was used as the extraction solvent for all samples at a concentration of 20 mg/mL. Briefly, accurately weighed samples were mixed with the corresponding extraction solvent and sonicated for 10 min (KH-700DE, Kunshan Hechuang Ultrasonic Instrument Co., Ltd., China; 100 kHz) to facilitate analyte release into the solvent phase. During sonication, the tubes were capped and immersed in the bath at near-room temperature to minimize solvent evaporation and heat accumulation. An ultrasonic bath rather than direct probe sonication was used because it allows sealed-tube operation, avoids direct contact between the probe and the organic extracts, reduces the risk of probe-derived contamination, and facilitates parallel processing of multiple samples under consistent conditions. The resulting suspensions were then subjected to high-speed centrifugation at 8000 rpm for 10 min to achieve solid–liquid separation. The clarified supernatants were carefully collected and directly used for subsequent GC–MS or LC–MS analysis.

### Magnetic particle-assisted solid–liquid separation procedure

As shown in Fig. 8B, plant-derived samples were transferred into centrifuge tubes, followed by the addition of an appropriate amount of Fe₃O₄ nanoparticles and the corresponding extraction solvent. The same sample-to-solvent ratios and ultrasonication time as those used in the conventional CSE were applied. The key difference lies in the introduction of magnetic nanoparticles, which enabled solid–liquid separation to be achieved by an external magnetic field after ultrasonication, without the need for centrifugation.

Specifically, for GC–MS analysis, EtOH was used as the extraction solvent for all samples except tobacco powder, which was extracted using DCM at a concentration of 50 mg/mL. For LC–MS analysis, MeOH was used as the extraction solvent for all samples at a concentration of 20 mg/mL. For powdered samples, a sample-to-particle mass ratio of 1:15 (w/w) was applied, whereas a ratio of 1:5 (w/w) was used for viscous extract samples. The mixtures were ultrasonicated for 10 min using an ultrasonic bath (KH-700DE, Kunshan Hechuang Ultrasonic Instrument Co., Ltd., China; 100 kHz) to promote analyte release into the solvent phase while facilitating the association of magnetic particles with insoluble solids. After extraction, an external magnetic field was applied to rapidly isolate the magnetic particle–solid composites, thereby achieving efficient solid–liquid separation. The clarified supernatants were carefully collected and directly subjected to subsequent GC–MS or LC–MS analysis.

### LC–MS and GC–MS analysis

LC–MS analyses were performed on a Thermo Fisher Exactive mass spectrometer coupled with a Thermo Scientific Ultimate 3000 ultra-high-performance liquid chromatography (UHPLC) system. Chromatographic separation was achieved using a Waters Acquity Premier BEH C18 column (100 mm × 2.1 mm, 1.7 μm). The mobile phases consisted of 0.1% (v/v) FA in water (mobile phase A) and ACN (mobile phase B). The flow rate was set at 0.3 mL/min, the column temperature was maintained at 35 °C, and the injection volume was 2 μL. The gradient elution program was as follows: 0 min, 15% B; 1.33 min, 30% B; 5.33 min, 38% B; 8 min, 43% B; 12 min, 45% B; 14 min, 50% B; 16.67 min, 60% B; 19.33 min, 60% B; 21.33 min, 90% B; 22.00 min, 100% B; 22.33 min, 100% B; 24.00 min, 15% B; and 27.00 min, 15% B. The mass spectrometer was operated in full-scan mode over a *m/z* range of 70–1000. Components detected by LC-MS were identified based on a comparison with data reported in the literature and the use of Compound Discoverer software (version 3.3, Thermo Fisher Scientific) for compound identification.

GC–MS analyses were conducted on an Agilent gas chromatography–mass spectrometry system equipped with a DB-5MS capillary column (60 m × 0.25 mm i.d., 0.25 μm film thickness). High-purity helium was used as the carrier gas at a constant flow rate of 1.0 mL/min. The oven temperature program was set as follows: initial temperature of 50 °C held for 4 min, increased to 280 °C at a rate of 5 °C/min, and then held at 280 °C for 10 min. Samples were injected in split mode (50:1) with an inlet temperature of 250 °C. The transfer line temperature was maintained at 280 °C. Electron ionization (EI) was employed at 70 eV, with the ion source and quadrupole temperatures set at 230 °C and 150 °C, respectively. The mass spectrometer was operated in full-scan mode over an *m/z* range of 50–500, with a solvent delay of 4.5 min. Components detected by GC–MS were identified by matching their mass spectra with entries in the National Institute of Standards and Technology (NIST) mass spectral library (NIST 20).

In the present study, GC-MS and LC-MS analyses were used primarily for qualitative chemical profiling and for relative comparison of extracts prepared using different pretreatment procedures, rather than for absolute quantification of selected target analytes. Accordingly, calibration curves were not constructed, and LOD and LOQ values for individual compounds were not determined.

## Supplementary information

Supplementary Material

## Data Availability

The datasets generated and/or analyzed during the current study are not publicly available because they contain raw chromatographic and mass spectrometric profiling data that are not deposited in a public repository, but are available from the corresponding author on reasonable request.
