# Supplementary material for: Magnetic particle-assisted solid–liquid separation: a rapid and centrifugation-free strategy for the analysis of plant-derived powders and viscous extracts
Source: NPJ Sci Food. 2026 Jul 10;10:340. doi: 10.1038/s41538-026-00980-8 (PMC13728144; doi:10.1038/s41538-026-00980-8)
Supplement: Supplementary file 1 [file 41538_2026_980_MOESM1_ESM.docx]

**Fig. S1.** SEM images of (A) pristine Fe₃O₄ particles, (B) pristine tobacco leaf powder particles, (C) Fe₃O₄/tobacco powder composite at higher magnification, and (D) Fe₃O₄/tobacco composite at lower magnification. The composites were obtained using the optimized MPAS procedure and vacuum-dried at 60 °C before SEM observation.

**Fig. S2.** SEM images of (A) pristine Fe₃O₄ particles, (B) pristine malt extract particles, (C) Fe₃O₄/malt extract composite at higher magnification, and (D) Fe₃O₄/malt extract composite at lower magnification. The composites were obtained using the optimized MPAS procedure and vacuum-dried at 60 °C before SEM observation.

**Fig. S3.** TICs of plant-derived sample extracts analyzed by GC–MS: (A) dandelion extract, (B) grape extract, (C) danshen powder, (D) nutgrass powder, (E) Scutellaria baicalensis powder, and (F) tobacco powder. Ethanol was used as the extraction solvent for most samples, while dichloromethane was used for tobacco powder.

**Fig. S4.** TICs of plant-derived sample extracts analyzed by high-resolution LC–MS in positive ion mode: (A) dandelion extract, (B) grape extract, (C) danshen powder, (D) nutgrass powder, (E) Scutellaria baicalensis powder, and (F) tobacco powder. Methanol was used as the extraction solvent, and MPAS was performed with 10 min ultrasonication followed by magnetic separation.

**Table S1.** Identification of major chemical constituents in different plant samples (malt extract, cinnamon powder, nutgrass powder, dandelion extract, and tobacco powder) by GC-MS analysis.

| Sample | Chemical Constituents | Molecular Formula | Retention time (min) |
| --- | --- | --- | --- |
| Malt extract | 2-Furanmethanol | C_5_H_6_O_2_ | 8.77 |
|  | 2-Hydroxy-3-Methyl-2-cyclopentenone | C_6_H_8_O_2_ | 13.63 |
|  | Maltol | C_6_H_6_O_3_ | 16.42 |
|  | 5-Hydroxymethylfurfural | C_6_H_6_O_3_ | 19.54 |
|  | Vanillin | C_8_H_8_O_3_ | 24.47 |
|  | Levoglucosan | C_6_H_10_O_5_ | 26.83 |
|  | 3,5-Dihydroxy-6-methyl-2H-pyran-4(3H)-one | C_6_H_8_O_4_ | 17.35 |
|  | 2-Hydroxy-4-methoxybenzaldehyde | C_8_H_8_O_3_ | 40.29 |
| Cinnamon powder | Cinnamaldehyde | C_9_H_8_O | 21.20 |
|  | α-Copaene | C_15_H_24_ | 24.04 |
|  | Coumarin | C_9_H_6_O_2_ | 25.74 |
|  | Trans-calamenene | C_15_H_22_ | 27.76 |
|  | 2-Methoxycinnamaldehyde | C_10_H_10_O_2_ | 27.84 |
|  | 1, 1, 5-Trimethyl-1, 2-dihydronaphthalene | C_13_H_16_ | 28.28 |
|  | Gleenol | C_15_H_26_O | 29.33 |
|  | Copaene | C_15_H_24_ | 30.75 |
|  | Palmitic acid | C_16_H_32_O_2_ | 37.02 |
|  | 9-Octadecenoic acid | C_18_H_34_O_2_ | 40.43 |
| Nutgrass powder | Calamenene | C_15_H_22_ | 23.78 |
|  | Trans-Calamenene | C_15_H_22_ | 24.12 |
|  | Cyperene | C_15_H_24_ | 24.89 |
|  | Rotundene | C_15_H_24_ | 26.41 |
|  | Caryophyllene oxide | C_15_H_24_O | 29.40 |
|  | Xylazine | C_12_H_16_N_2_S | 30.27 |
|  | 9H-Cycloisolongifolene, 8-oxo- | C_15_H_22_O | 30.36 |
|  | Alloaromadendrene oxide | C_15_H_24_ | 30.64 |
|  | α-Cyperone | C_15_H_22_O | 33.06 |
| Dandelion extract | Methylthioethane | C_3_H_8_S | 6.44 |
|  | 2-Furanmethanol | C_5_H_6_O_2_ | 8.64 |
|  | 5-Hydroxymethylfurfural | C_6_H_6_O_3_ | 19.49 |
|  | 2-Cyanobenzaldehyde | C_8_H_5_NO | 22.70 |
|  | Lethane | C_9_H_17_NO_2_S | 25.39 |
|  | D-Allose | C_6_H_12_O_6_ | 26.80 |
|  | D-altronic acid | C_6_H_12_O_7_ | 29.16 |
| Tobacco powder | 2,4-Dimethylheptane | C_9_H_20_ | 7.81 |
|  | 3-Ethyl-3-methylheptane | C_10_H_22_ | 14.37 |
|  | 3,5-Di-tert-butyltoluene | C_15_H_24_ | 20.27 |
|  | L-Nicotine | C_10_H_14_N_2_ | 23.20 |
|  | 2,4-Di-t-butylphenol | C_14_H_22_O | 27.09 |
|  | Phytyl acetate | C_22_H_42_O_2_ | 34.58 |
|  | n-Hexadecanoic acid | C_16_H_32_O_2_ | 37.03 |
|  | Octadecane | C_18_H_38_ | 50.32 |

**Table S2.** Identification of major chemical constituents in different plant samples (malt extract, cinnamon powder, nutgrass powder, dandelion extract, and tobacco powder) by high-resolution LC-MS analysis.

| Sample | Chemical Constituents | Molecular Formula | Theoretical *m/z* [M+H]^+^ | Measured *m/z* [M+H]^+^ | Mass deviation (ppm) | Retention time (min) |
| --- | --- | --- | --- | --- | --- | --- |
| Malt extract | Epigallocatechin-3-gallate | C_22_H_18_O_11_ | 459.0921 | 459.0916 | 1.2 | 1.47 |
|  | Maltol | C_6_H_6_O_3_ | 127.0389 | 127.0390 | 0.5 | 1.72 |
|  | Glutaric acid | C_5_H_8_O_4_ | 133.0495 | 133.0495 | -0.4 | 0.92 |
|  | Isoleucine | C_6_H_13_NO_2_ | 132.1019 | 132.1019 | -0.2 | 0.93 |
|  | Betaine | C_5_H_11_NO_2_ | 118.0862 | 118.0864 | 1.4 | 0.88 |
|  | Vanillic acid | C_8_H_8_O_4_ | 169.0495 | 169.0494 | 1.1 | 3.79 |
|  | Vanillin | C_8_H_8_O_3_ | 153.0546 | 153.0544 | -1.6 | 3.24 |
|  | t-Ferulic acid | C_10_H_10_O_4_ | 195.0651 | 195.0650 | 1.0 | 3.43 |
|  | Sinapinic acid | C_11_H_12_O_5_ | 225.0757 | 225.0755 | 1.2 | 0.91 |
|  | p-Hydroxybenzoic acid | C_7_H_6_O_3_ | 139.0389 | 139.0386 | 2.5 | 2.77 |
| Cinnamon powder | β-Pinene | C_10_H_16_ | 137.1325 | 137.1322 | -1.8 | 19.05 |
|  | p-Cymene | C_10_H_14_ | 135.1168 | 135.1166 | -1.6 | 21.64 |
|  | Borneol acetate | C_12_H_20_O_2_ | 197.1536 | 197.1533 | -1.5 | 4.60 |
|  | 6,6-Dimethyl-2-methylidenebicyclo[3.1.1]heptan-3-ol | C_10_H_16_O | 153.1273 | 153.1272 | -1.3 | 10.18 |
|  | 4-(1-Methylethyl)-2-cyclohexen-1-one | C_9_H_14_O | 139.1117 | 139.1114 | -1.6 | 21.66 |
|  | 2,2,5a,9-Tetramethyloctahydro-2H-3,9a-methanobenzo[b]oxepine | C_15_H_26_O | 223.2056 | 223.2050 | -2.6 | 23.48 |
|  | Cuminaldehyde | C10H_12_O | 149.0960 | 149.0958 | -1.7 | 19.05 |
|  | α-Calacorene | C_15_H_20_ | 201.1637 | 201.1634 | -1.4 | 19.25 |
|  | 4a,8-Dimethyl-hexahydronaphthalen-2(3H)-one | C_12_H_18_O | 179.1430 | 179.1426 | -2.1 | 20.58 |
| Nutgrass powder | D-Limonene | C_10_H_16_ | 137.1324 | 137.1321 | 2.9 | 19.04 |
|  | p-Cymene | C_10_H_14_ | 135.1168 | 135.1165 | 1.9 | 3.76 |
|  | p-Cymenene | C_10_H_12_ | 133.1011 | 133.1009 | 1.7 | 21.43 |
|  | (+)-Dihydrocarvone | C_10_H_16_O | 153.1273 | 153.1270 | 2.2 | 5.95 |
|  | Dihydro-carvylacetate | C_12_H_20_O_2_ | 197.1536 | 197.1532 | 2.1 | 10.54 |
|  | Carvone | C10H14O | 151.1117 | 151.1114 | 2.1 | 8.01 |
|  | 1-α-Terpineol | C_10_H_18_O | 155.1430 | 155.1428 | 1.3 | 10.27 |
|  | 6β-Hydroxyipolamiide | C_17_H_26_O_12_ | 423.1497 | 423.1456 | 9.6 | 0.84 |
|  | Isocryptomerone | C_15_H_24_O_2_ | 237.1849 | 237.1842 | 2.9 | 13.48 |
|  | α-Cyperol | C15H24O | 221.1899 | 221.1892 | 3.1 | 21.14 |
| Dandelion extract | Gallic acid | C_7_H_6_O_5_ | 171.0287 | 171.0280 | -0.4 | 1.51 |
|  | Chlorogenic acid | C_16_H_18_O_9_ | 355.1023 | 355.1022 | 0.2 | 1.27 |
|  | Apigenin-8-C-glucoside | C_21_H_20_O_10_ | 433.1129 | 433.1128 | 0.2 | 3.78 |
|  | Liquiritigenin | C_15_H_12_O_4_ | 257.0808 | 257.0807 | 0.4 | 3.14 |
|  | Luteolin | C_15_H_10_O_6_ | 287.0550 | 287.0549 | 0.3 | 3.57 |
|  | Quercetin | C_15_H_10_O_7_ | 303.0499 | 303.0498 | 0.3 | 3.15 |
|  | Kaempferide | C_16_H_12_O_6_ | 301.0707 | 301.0706 | 0.1 | 3.95 |
|  | Neochlorogenic acid | C_16_H_18_O_9_ | 355.1023 | 355.1022 | 0.1 | 1.27 |
|  | Isoorientin | C_21_H_20_O_11_ | 449.1078 | 449.1082 | -0.7 | 3.23 |
|  | Rutin | C_27_H_30_O_16_ | 611.1606 | 611.1610 | -0.6 | 4.64 |
|  | Hesperidin | C_28_H_34_O_15_ | 611.1970 | 611.1964 | 1.0 | 3.9 |
|  | Isohyperoside | C_21_H_20_O_12_ | 465.1027 | 465.1027 | -0.1 | 3.16 |
| Tobacco powder | Chlorogenic acid | C_16_H_18_O_9_ | 355.1023 | 355.1014 | 2.5 | 1.57 |
|  | Caffeic acid | C_9_H_8_O_4_ | 181.0495 | 181.0491 | 2.2 | 2.21 |
|  | Ferulic acid | C_10_H_10_O_4_ | 195.0651 | 195.0645 | 3.0 | 3.43 |
|  | Rutin | C_27_H_30_O_16_ | 611.1606 | 611.1589 | 2.8 | 2.96 |
|  | Scopoletin | C_10_H_8_O_4_ | 193.0495 | 193.0491 | 1.7 | 3.40 |
|  | Scopolin | C_16_H_18_O_9_ | 355.1023 | 355.1014 | 2.6 | 1.57 |
|  | Coumarin | C_9_H_6_O_2_ | 147.0440 | 147.0437 | 1.9 | 2.23 |
|  | Kaempferitrin | C_27_H_30_O_14_ | 579.1708 | 579.1694 | 2.4 | 4.01 |
|  | Kaempferol-3-O-rutinoside | C_27_H_30_O_15_ | 595.1657 | 595.1651 | 0.9 | 3.38 |
|  | Kaempferol | C_15_H_10_O_6_ | 287.0550 | 287.0540 | 3.2 | 3.38 |
